# Seroprevalence of HIV, HBV, HCV and Syphilis among blood donors in a Nigerian tertiary medical centre

**DOI:** 10.1186/s12879-025-11024-z

**Published:** 2025-04-30

**Authors:** Ismail Habibu, Bashir Mohammed Abubakar, Ibrahim Musa Moi, Rabiu Abdulrazaq

**Affiliations:** 1 Department of Biological Sciences, Bauchi State University Gadau, PMB 065, Bauchi, Nigeria; 2 Present Address: Department of Microbiology, Bauchi State University Gadau, PMB 065, Bauchi, Nigeria; 3 Department of Laboratory Services, Federal Medical Centre, PMB 005, Azare, Bauchi, Nigeria

**Keywords:** Transfusion-transmissible infection, HIV, HBV, HCV, Syphilis, Blood donors, Nigeria

## Abstract

**Background:**

Transfusion-transmitted infections (TTIs), such as HIV, HBV, HCV, and Syphilis, present considerable difficulties in maintaining blood quality despite the critical role of blood transfusion in emergency medical care. This study aims to ascertain the prevalence of these infections and the factors that increase their risk among those who donate blood.

**Methods:**

A cross-sectional study was conducted at the Federal Medical Center in Azare, involving 400 blood donors. Serological tests were conducted for HBV, HCV, HIV, and syphilis, and sociodemographic data was collected through a structured questionnaire. The univariate and multivariate logistic regression tests were employed to detect associated risk factors, with a significance level set at *P* < 0.05.

**Result:**

Of the total blood donors, 17.00% (68/400) tested positive for at least one TTIs. The presence of HIV, HBV, HCV, and syphilis was identified in 2.8% (11/400), 8.3% (33/400), 1.8% (7/400), and 4.3% (17/400) of the donors, respectively. Multivariate analysis, after adjustments with various variables, indicates only commercial blood donors [Adjusted Odds Ratio (AOR) (95% CI): 14.63 (1.76-121.27)] and multiple sexual partners [AOR (95% CI): 5.40 (1.28–22.70)] were associated with HIV, while blood transfusion and piercing or tattoo were associated with HBV. Multiple sexual partners and a history of STDs were associated with syphilis infection.

**Conclusion:**

TTIs such as HIV, HBV, HCV, and syphilis were detected among the blood donors, with HBV being the most common. The findings highlight a gender disparity in blood donation, with voluntary donors comprising the majority; nevertheless, commercial donors had the highest prevalence of TTIs. Enhanced donor screening and public awareness are crucial for blood safety.

**Clinical trial number:**

Not applicable.

**Supplementary Information:**

The online version contains supplementary material available at 10.1186/s12879-025-11024-z.

## Introduction

A blood transfusion may be necessary to save life, as millions rely on blood transfusions to recover from blood loss, given the absence of an artificial substitute [1]. The World Health Organization (WHO) reports that approximately 118.54 million blood donations are collected globally [2]. However, ensuring the global availability of safe blood and blood products remains a significant challenge due to the risk of transmitting harmful transfusion-transmissible infections (TTIs), such as Human Immunodeficiency Virus (HIV), Hepatitis B Virus (HBV), Hepatitis C Virus (HCV), and syphilis. The primary factor in transmitting these infectious agents through blood transfusions is the presence of blood-borne pathogens in the blood cells of asymptomatic donors [3]. These TTIs can have long-term detrimental effects on recipients, their families, and communities. To mitigate this risk, the WHO recommends rigorous screening of all blood donations for HIV, HBV, HCV, and syphilis before transfusion [2].

The WHO reported that by 2023, 39.9 million people were living with HIV, with Africa having the highest prevalence, affecting 3.4% of the adult population. Women and girls accounted for 62% of newly acquired HIV infections [4]. In 2015, viral hepatitis caused 1.34 million fatalities globally, with 96% caused by complications from persistent HBV and HCV infections [5]. Hepatitis B virus is highly contagious, transferring even without visible blood and surviving on surfaces for at least one week [6]. In 2024, over 50 million people worldwide suffer from chronic HCV infection, with 1.0 million new infections per year [5]. The Eastern Mediterranean Region has the highest disease burden, with 12 million chronically sick, while eight million are persistently infected in the African Region [7].

Syphilis is an infectious disease that affects the entire body and is caused by the *Treponema pallidum*. The transmission of the disease can occur through sexual intercourse, blood transfusion, and from mother to child during pregnancy [8]. Syphilis remains a prominent public health concern in sub-Saharan Africa. The incidence of persistent syphilis cases in African countries exhibited variation, as seen by studies conducted in Nigeria [9] and Sudan [10], reporting prevalence rates of 3.10%, 11.90%, and 2.10%, respectively.

A study conducted in 2020 aimed to ascertain the prevalence of HIV, HBV, HCV, and syphilis among blood donors in Ethiopia. The study reported the following prevalence rates: HIV at 0.4%, HBV at 0.40%, HBV at 2.40%, and syphilis at 0.90% [11]. HCV affects 130–150 million people globally, with Africa accounting for 8% of infections, and approximately 500,000 people die annually from HCV-related liver diseases [12]. Implementing a pre-donation screening system for blood donors can effectively decrease the prevalence rates of HIV, HBV, and HCV. Furthermore, providing donors with information regarding HBV and HCV transmission routes may enhance their understanding of the importance of minimizing risk factors [13, 14].

Sub-Saharan Africa faces significant public health challenges due to insufficient national blood transfusion policies and infrastructure, underqualified staff, limited financial resources, and concerns about blood safety. Nigeria’s National Blood Transfusion Service (NBTS) was established in 2006 to address these issues. The system includes various levels such as the zonal blood service centres, State and Local Government Areas blood service centres, armed forces blood service centres, private health organizations, and other non-governmental health organizations [9]. However, Nigeria still lacks political determination and receptiveness to new ideas for improving blood safety and accessibility through voluntary donors.

TTIs represent a substantial global concern, threatening blood safety and public health. The transfusion of blood plays a crucial role in healthcare services. The WHO reports that out of the total blood donations collected worldwide, 58% originate from low- and middle-income countries [2]. Although blood donation can enhance patients’ quality of life, it remains a primary source for transmitting infectious pathogens.

While these global issues are critical, their implications are particularly pronounced in Nigeria. The country’s prevalence of TTIs among blood donors remains higher and is also one of the most serious complications of blood transfusion [9]. For instance, the Polaris Observatory Collaborators conducted a study across 128 countries, revealing a mean global HBV prevalence of 4.9%, with Nigeria and other Asian nations, including China, India, Indonesia, and the Philippines, representing over 57% of all HBsAg positive cases [9]. This figure highlights the immediate need for focused study and initiatives specific to the Nigerian context.

Thus, despite the significant occurrence of TTIs among blood donors reported from various parts of Nigeria, there is a paucity of information regarding the frequency of these infections in the present study area. The data collected will help evaluate preventive strategies and corrective actions. Therefore, the data acquired from the present investigation will allow us to ascertain the health risks of giving blood for transfusion. This will facilitate the implementation of improved screening procedures, guaranteeing the safety and sufficiency of blood donations for transfusion. The study aims to determine the seroprevalence of HIV, HBV, HCV, and *Treponema pallidum* among blood donors at the Federal Medical Centre (FMC) Azare. The data will help evaluate the safety of specific blood for transfusion and potentially enable health policymakers to implement an improved diagnostic system.

## Materials and methods

### Study design and study population

This research is a cross-sectional investigation carried out from January 2024 to June 2024 among individuals donating blood to the blood bank of the Federal Medical Centre (FMC), Azare, Nigeria. The medical Centre is a tertiary facility owned by the Federal Government of Nigeria. Azare, located in the Katagum division, serves as the headquarters of the Katagum division in Bauchi State. The Ethical Committee of FMC Azare approved the study in December 2023 with the reference ID of FMCA/COM/35/VOL1. All the prospective blood donors were explained the purpose of the study and enrolled after signing the informed consent.

### Inclusion and exclusion criteria

The study encompassed all blood donors who visited the blood bank in the study area during the data collection period, meeting the national blood donation criteria, which included being 18 years or older, donating blood at least once in the years preceding the study, having a body weight exceeding 50 kg, and signing the consent form. Exclusions were applied to study participants who were either unwilling to provide consent or deemed not mentally fit.

### Sample size

The sample size for the current study was calculated using standard formula [15]. The study area did not possess any available prevalence estimates for HIV, HBV, HCV, and syphilis among blood donors. Thus, to get a minimum sample size, a 50% prevalence rate for HIV, HBV, HCV, and syphilis among blood donors was used as prior or existing data were absent for TTIs in the study area.

$$\:N=\frac{{Z}^{2}pq}{{d}^{2}}$$

Where Zα/2 represents the number corresponding to a 95% confidence level, and p represents the assumed proportion of HIV, HBV, HCV, and syphilis among blood donors. *q* = 1 *−* Assumed proportion of HIV, HBV, HCV, and syphilis among blood donors, and d represents the margin of error in calculating this proportion, p.

$$\:n=\frac{{\left(1.96\right)}^{2}*0.5*0.5}{{\left(0.05\right)}^{2}}$$

$$\:n=\frac{0.9604}{0.0025}$$

$$\:n=384.16$$

A minimum sample size of 384.16 was determined. Nevertheless, the calculated sample size was modified to 400, which served as the baseline sample size for our investigation to prevent any bias in study participant selection.

### Data collection

A structured questionnaire was administered to the study participants. The questionnaire was developed from a previous literature review and used as a research tool. An expert in the original survey verified the content validity, and it was supported by further recent research [9, 11, 19]. The questionnaire gathers information on the participants’ demographic characteristics and the risks associated with transmitting TTIs among blood donors. The sociodemographic characteristics include the blood donors’ age, sex, marital status, level of education, occupation, marital status, and residential area. The risk characteristics included in the questionnaire are factors such as surgical history, history of blood transfusion, multiple sexual partners, history of STDs, sharing of a razor or sharp material, frequency and type of donation, piercing/ tattoo, tribal marks, and circumcision. After the donors agreed to participate in the study, informed consent was obtained from the prospective blood donors, and data were anonymized before analysis.

### Sample collection and Preparation

Five millimetres (5 ml) of blood samples were aseptically collected from the prospective blood donors using a sterile syringe and transferred into a clean anticoagulant container that contained Ethylenediaminetetraacetic acid (EDTA). The samples were subsequently centrifuged at 3,000 rpm for 5 min at room temperature, and the serum portion was used as a test strip for antigen or antibody detection.

Antibody to HIV infection was detected using the HIV rapid test kits Determine^®^ HIV 1/2 strips (Abbott Diagnostic Medical Co. Ltd., Japan; Batch No: 0000692576). These rapid test kits have a specificity of 99% and sensitivity of 98% and were used to detect HIV type 1 (HIV-1) and (HIV-2) antibodies, according to the manufacturer’s instructions. The strip has two horizontal lines labelled “control and “patient” bars. A single red line on the strip at position C (control) indicated a reasonable control. A red line in the patient bar indicated a positive result for HIV-1 or HIV-2, whereas its absence signified a negative result. The non-reactive samples using the Determine^®^ HIV 1/2 strips are reported as negative, while reactive (positive) results undergo confirmation tests to verify the results.

The confirmation test was performed using Uni-Gold™ Recombigen^®^ (Trinity Biotech, Ireland; Batch No: 2300098) and Stat-Pak (Chembio Diagnostic Systems, NY, USA; Batch No:44081722). First, positive samples were subjected to confirmatory using the Uni-Gold kit, which has a relative sensitivity and specificity of 100% according to the manufacturer’s instructions. Reactive samples were reported as positive and were directed for counselling. In contrast, samples with negative results (considered discordant) were verified using a tie-breaker Stat-pak, which has a relative sensitivity and specificity of 100%. Briefly, two drops of blood collected were deposited into the sample port of the Uni-Gold device. Two drops of running buffer were subsequently applied to the sample port. The outcome was red after ten minutes. A “reactive” result signifies that the individual who provided the blood is HIV-positive, whereas a “non-reactive” result denotes that they are HIV-negative. The Stat-Pak was utilized as a tie-breaker. This is a rapid point-of-care test for identifying HIV-1 and HIV-2 antibodies in fingerstick whole blood, whole venous blood, serum, or plasma. The interpretation of Stat-Pak parallels that of Uni-Gold.

**Fig. 1:**
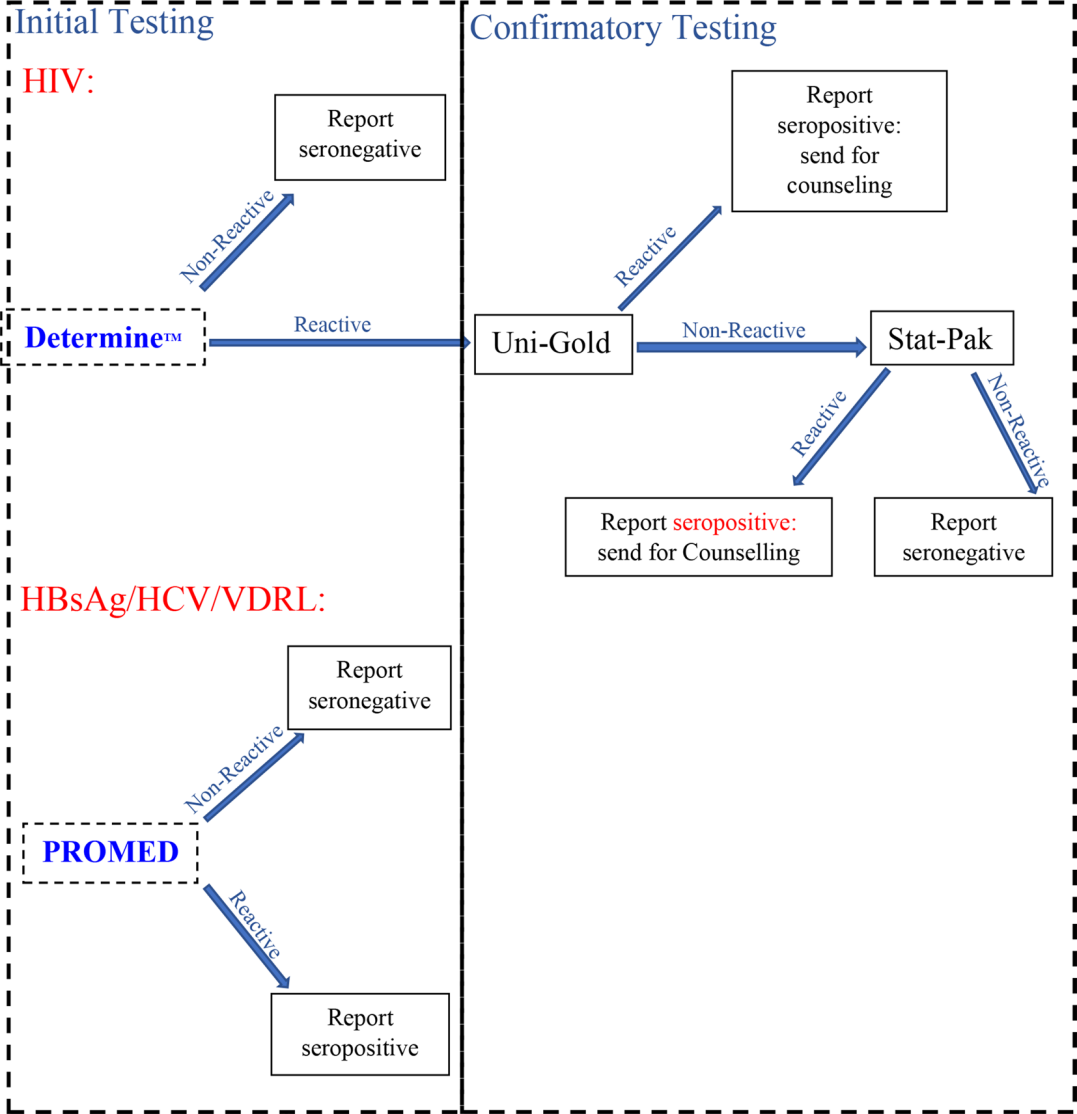
Algorithm for serological screening for blood donors

The presence of HBsAg in serum was detected using PROMED rapid test kits (Xinghu Co. Ltd, China; Batch No: 24022011301), with a relative sensitivity of 100% and specificity of 99%. The Anti HCV was detected using PROMED rapid test kits (Xinghu Co. Ltd, China; Batch No: 20240820), with a relative sensitivity and specificity of 99% following the manufacturer’s instructions. Finally, the VDRL was detected using the PROMED rapid test kits (Xinghu Co. Ltd, China; Batch No: 24070102), which have a sensitivity and specificity of 100% and 99.58% according to the manufacturer’s instructions. The detailed algorithm used to process the blood samples is shown in Fig. 1.

### Quality assurance

The principal investigator regularly monitored the data collectors to ensure completeness of data. All diagnostic kits in the present study have been checked for proper storage conditions and expiration dates. Quality control of the serological test involved running known positive and negative controls parallel with the test samples. All laboratory operations were conducted per standard operating procedures (SOPs), and the manufacturer’s instructions were strictly followed for each reagent lot.

### Data analysis

Following data gathering, the questionnaires underwent a comprehensive examination, manually cleaned, assigned codes, and inputted into a Microsoft™ Excel spreadsheet (MS Office Excel^®^ 2016). The data was imported into SPSS software version 27 (IBM) for subsequent analysis. The participant’s attributes were quantitatively described using statistical measures such as means and frequencies given as percentages (%). The variables related to HIV, HBV, HCV, and syphilis infection, together with their associated risk factors, were evaluated using univariate and multivariate logistic regression. Variables with a p-value lower than 0.05 in the univariate logistic regression model were then incorporated into the multivariate logistic regression model. The variables’ significance level and association were examined using a 95% Confidence Interval (CI) and odds ratios (ORs). The statistical analysis will assess the variables’ importance and relationship using a 95% Confidence Interval (CI) and Odds ratios (ORs). P-values less than 0.05 were deemed to be statistically significant. The Hosmer and Lemeshow test evaluated the model’s adequacy at a significance level of 0.05.

## Results

### Sociodemographic characteristics

Of the total blood donors, 99.00% (396/400) were males, and 1.00% (4/400) were females. The mean age of the individuals in the study was 29.33 ± 6.91, and 63.00% (252/400) of the blood donors were in a marital relationship. The majority of participants in the present study possess a secondary level of education. Approximately 42.75% (171/400) of blood donors are employed in business professions, accounting for almost half the total. Furthermore, most of them reside in the urban area 63.75% (255/400) (Table 1).

**Table 1:** Sociodemographic characteristics of 400 blood donors at FMC, Azare Northeast Nigeria

Characteristics	*n* (%)
**Gender**	
Male	396 (99.00)
Female	4 (1.00)
**Age group**	
18–27 years	187 (46.75)
28–37 years	157 (39.25)
38–47 years	52 (13.00)
> 48 years	4 (1.00)
**Marital status**	
Single	140 (35.00)
Married	252 (63.00)
Divorced	8 (2.00)
**Educational level**	
Cannot read and write	24 (6.00)
Primary Education	83 (20.75)
Secondary Education	218 (54.50)
Tertiary	74 (18.50)
**Occupation**	
Civil servant	26 (6.50)
Student	62 (15.50)
Farmer	83 (20.75)
Business	171 (42.75)
Teaching	27 (6.75)
Driving	27 (6.75)
Housewife	4 (1.00)
**Resident**	
Urban	255 (63.75)
Rural	145 (36.25)

### TTIs prevalence among blood donors

The percentage of blood donors infected with at least one TTIs marker was 17.00%; 68/400 (95% Confidence Interval (CI):13.40–21.00). The prevalence of HIV among these study participants was 2.80%; 11/400 (95% Confidence interval (CI): 1.40–4.90), HBV was 8.20%; 33/400 (95% Confidence Interval (CI): 5.70–11.40), HCV 1.80%; 7/400 (95% Confidence Interval (CI): 0.70–3.60) and syphilis infection was 4.20%; 17/400 (95% Confidence Interval (CI): 2.50–6.70) (Table 2). Males exhibit a higher rate of seropositivity for HIV, HBV, HCV, or syphilis compared to females, potentially attributed to the male predominance among the study participants who donated blood. The prevalence of HIV, HBV, HCV, and syphilis is higher in younger age groups (18–27 and 28–37) compared to older age groups (38–47 and 48 years and above) as shown in Table 3.

**Table 2:** Prevalence of HIV, HBV, HCV, and syphilis among 400 blood donors at FMC, Azare Northeast Nigeria

Variable	Category	Number	Prevalence (%)	95% CI
HIV	Positive	11	2.80	1.40–4.90
	Negative	389		
HBV	Positive	33	8.20	5.70–11.40
	Negative	367		
HCV	Positive	7	1.80	0.70–3.60
	Negative	393		
Syphilis	Positive	17	4.20	2.50–6.70
	Negative	383		
Overall TTIs	Positive	68	17.00	13.40–21.00
	Negative	332		

CI = Confidence interval

**Table 3:** Sociodemographic characteristics between HIV, HBV, HCV, and syphilis infected and non-infected among 400 blood donors at FMC, Azare Northeast Nigeria

Variables	HIV Pos. *N* (%)	HBV Pos. *N* (%)	HCV Pos. *N* (%)	Syphilis Pos. *N* (%)
**Gender**				
Male	7 (1.80)	33 (8.30)	7 (1.80)	17 (4.29)
Female	0 (0.00)	0 (0.00)	0 (0.00)	0 (0.00)
*p-value*	0.74	0.55	0.79	0.67
**Age group**				
18–27 years	4 (2.10)	19 (10.20)	3 (1.60)	3 (1.60)
28–37 years	6 (3.80)	12 (7.60)	4 (2.50)	13 (8.30)
38–47 years	1 (1.90)	2 (3.80)	0 (0.00)	1 (1.90)
> 48 and above	0 (0.00)	0 (0.00)	0 (0.00)	0 (0.00)
*p-value*	0.76	0.45	0.66	0.02
**Marital status**				
Single	3 (2.10)	12 (8.60)	1 ((0.70)	5 (3.60)
Married	8 (3.20)	21 (8.30)	6 (2.40)	12 (4.80)
Divorced	0 (0.00)	0 (0.00)	0 0 (0.00)	0 (0.00)
*p-value*	0.75	0.69	0.45	0.71
**Level of educational**				
Cannot read and write	0 (0.00)	1 (4.20)	1 (4.20)	1 (4.20)
Primary Education	6 (7.20)	4 (4.80)	1 (1.20)	4 (4.80)
Secondary Education	3 (1.40)	26 (11.90)	4 (1.80)	11 (5.00)
Tertiary	2 (2.70)	2 (2.70)	1 (1.40)	1 (1.40)
*p-value*	0.04	0.03	0.79	0.59
**Occupation**				
Civil servant	1 (3.80)	1 (3.80)	1 (3.80)	2 (7.70)
Student	2 (3.20)	4 (6.50)	1 (1.60)	3 (4.80)
Farmer	3 (3.60)	7 (8.40)	4 (4.80)	5 (6.00)
Business	4 (2.30)	17 (9.90)	0 0 (0.00)	6 (3.50)
Teaching	0 (0.00)	2 (7.40)	1 (3.70)	0 (0.00)
Driving	1 (3.70)	2 (7.40)	0 (0.00)	1 (3.70)
Housewife	0 (0.00)	0 (0.00)	0 (0.00)	0 (0.00)
*p-value*	0.96	0.92	0.15	0.79
**Resident**				
Urban	7 (2.70)	22 (8.60)	3 (1.20)	13 (5.10)
Rural	4 (2.80)	11 (7.60)	4 (2.80)	4 (2.80)
*p-value*	0.99	0.72	0.25	0.27

### Co-infection prevalence

According to the current study, 1.00% (4/400) of the study participants in the present study were co-infected with either HIV, HBV, HCV, or syphilis. Among the participants with co-infections, the following patterns were observed exclusively in males: HIV/syphilis (0.50%, 2/400), HBV/syphilis (0.25%, 1/400), and HCV/syphilis (0.25%, 1/400). Except for HBV/syphilis, all of the co-infection blood donors were 28 to 37 years old. 50.00% of the co-infected study participants were commercial donors.

### Risk factors of TTIs in individuals that donate blood

**Fig. 2:**
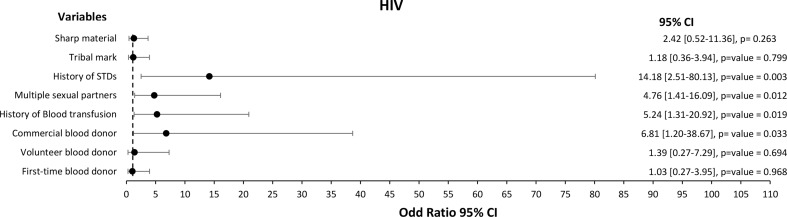
Forest plot of the risk factors associated with HIV among 400 blood donors at FMC, Azare Northeast Nigeria

We performed univariate and multivariate logistic regression analyses to assess the independent association between the risk factors and the occurrence of HIV, HBV, HCV, or syphilis infections. The univariate analysis demonstrated that the risk factors associated with HIV infection among the study participant were significantly associated with commercial blood donors [Crude Odd Ratio (COR) (95% CI): 6.81 (1.20-38.67)], history of blood transfusion [COR (95% CI): 5.24 (1.31–20.92)], multiple sexual partners [COR (95% CI): 4.76 (1.41–16.09)] and history of STDs [COR (95% CI): 14.18 (2.51–80.13)] (Fig. 2).

The multivariate model was fitted, and only commercial blood donors [Adjusted Odds Ratio (AOR) (95% CI): 14.63 (1.76-121.27)] and multiple sexual partners [AOR (95% CI): 5.40 (1.28–22.70)] were significantly associated with HIV (Table 4).

**Table 4:** Univariate and multivariate logistic regression of the risk factors associated with HIV among 400 blood donors at FMC, Azare Northeast Nigeria

Characteristics	Total	HIV Pos. *N* (%)	COR (95% CI)	*p*-value	AOR (95% CI)	*p*-value
**History of blood donation**						
First time	107	3 (2.80)	1.03 (0.27–3.95)	0.968		
Repeated	293	8 (2.70)	1	1		
**Types of blood donor**						
Parental	128	2 (1.60)	1			
Volunteer	231	5 (2.20)	1.39 (0.27–7.29)	0.694	2.95 (0.44–19.74)	0.265
Commercial donor	41	4 (9.80)	6.81 (1.20-38.67)	0.033	14.63 (1.76-121.27)	0.013
**Surgical history**						
No	377	11 (2.90)	1			
Yes	23	0 (0.00)	0.0 (0.00)	0.999		
**History of blood transfusion**						
No	371	8 (2.20)	1		1	
Yes	29	3 (10.30)	5.24 (1.31–20.92)	0.019	4.77 (0.85–26.75)	0.076
**Multiple sexual partners**						
No	337	6 (1.80)	1			
Yes	63	5 (7.90)	4.76 (1.41–16.09)	0.012	5.39 (1.28–22.70)	0.022
**History of STD**						
No	392	9 (2.30)	1			
Yes	8	2 (25.00)	14.18 (2.51–80.13)	0.003	5.70 (0.60-53.79)	0.128
**Piecing and tattoo**						
No	391	11 (2.80)	1			
Yes	9	0 (0.00)	0.00 (0.00)	0.999		
**Tribal marks**						
No	198	5 (2.50)	1			
Yes	202	6 (3.00)	1.18 (0.36–3.94)	0.799		
**Circumcision**						
No	5	0	1			
Yes	395	11 (2.80)	0.00 (0.00)	0.999		
**Sharp material**						
No	138	2 (1.40)	1			
Yes	262	8 (3.40)	2.42 (0.52–11.36)	0.263		

COR: Crude Odd Ratio; AOR: Adjusted Odds Ratio; 95% CI: 95% Confidence Interval

**Fig. 3:**
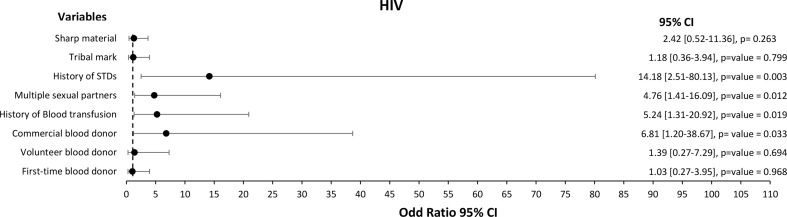
Forest plot of the risk factors associated with HBV among 400 blood donors at FMC, Azare Northeast Nigeria

The univariate analysis showed a significant association between HBV and a history of blood transfusion [COR (95% CI): 5.27 (2.12–13.09)], piercing or tattoo [COR (95% CI): 6.02 (1.43–25.27)], and tribal mark [COR (95% CI): 2.29 (1.11–5.16)] (Fig. 3).

The multivariate model was fitted, and only history of blood transfusion [AOR (95% CI): 4.33 (1.64–11.39)] and piercing or tattoo [AOR (95% CI): 5.02 (1.11–22.28)] were substantially linked to HBV (Table 5).

**Table 5:** Univariate and multivariate logistic regression of the risk factors associated with HBV among 400 blood donors at FMC, Azare Northeast Nigeria

Characteristics	Total	HBV Pos. *N* (%)	COR (95% CI)	*p*-value	AOR (95% CI)	*p*-value
**History of blood donation**						
First-time	107	7 (6.50)				
Repeated	293	26 (8.90)	1.39 (0.59–3.33)	0.455		
**Types of blood donor**						
Parental	128	11 (8.60)	1.19 (0.32–4.49)	0.797		
Volunteer	231	19 (8.30)	1.14 (0.32–4.03)	0.844		
Commercial donor	41	3 (7.30)	1			
**Surgical history**						
No	377	28 (7.40)	1			
Yes	23	5 (21.70)	3.46 (1.19–10.02)	0.022		
**History of blood transfusion**						
No	371	25 (6.70)	1		1	
Yes	29	8 (27.60)	5.27 (2.12–13.09)	0.001	4.33 (1.64–11.39)	0.003
**Multiple sexual partners**						
No	337	26 (7.70)	1			
Yes	63	7 (11.10)	1.14 (0.62–3.61)	0.371		
**History of STD**						
No	392	33 (8.40)	1			
Yes	8	0 (0.00)	0.00 (0.00)	0.999		
**Piercing or tattoo**						
No	391	30 (7.70)	1		1	
Yes	9	3 (33.30)	6.02 (1.43–25.27)	0.014	5.02 (1.11–22.28)	0.036
**Tribal mark**						
No	197	10 (5.10)	1			
Yes	203	23 (11.30)	2.29 (1.11–5.16)	0.027		
**Circumcision**						
No	5	1 (20.00)	1			
Yes	395	32 (8.10)	2.84 (0.31–26.14)	0.368		
**Sharp material**						
No	138	7 (5.10)	2.06 (0.87–4.88)	0.100		
Yes	262	26 (9.90)				

COR: Crude Odd Ratio; AOR: Adjusted Odds Ratio; 95% CI: 95% Confidence Interval

**Fig. 4:**
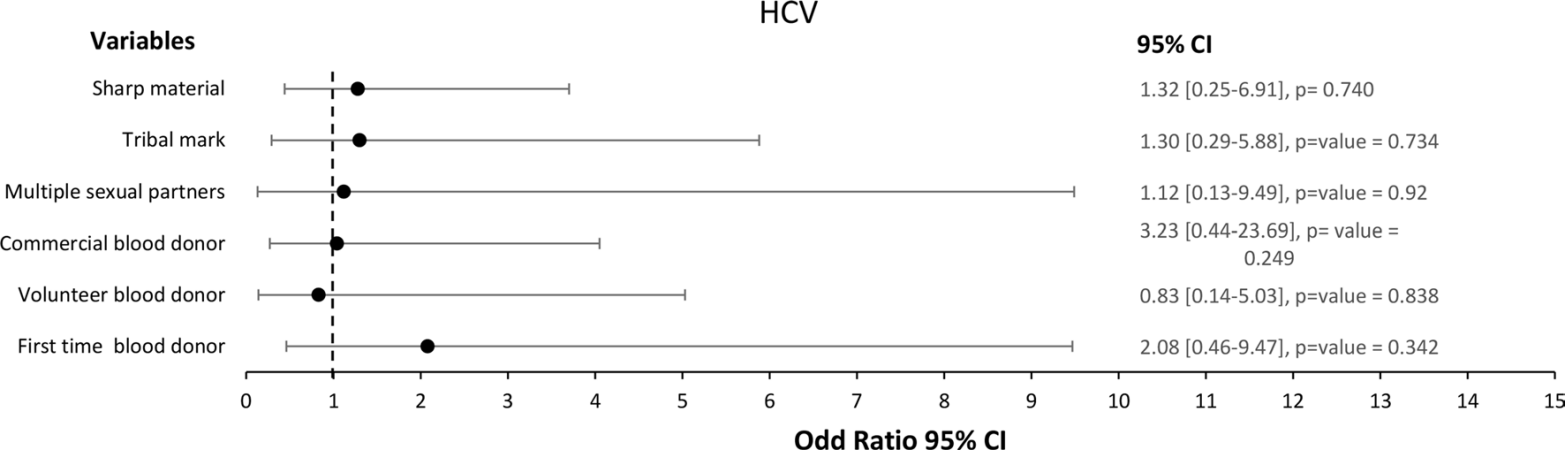
Forest plot of the risk factors associated with HCV among 400 blood donors at FMC, Azare Northeast Nigeria

The univariate analysis demonstrated that none of the risk factors of TTIs among blood donors is significantly associated with HCV (Fig. 4). Table 6 also shows the non-statistically significant HCV difference among individuals who donated blood in the study area.

**Table 6:** Univariate and multivariate logistic regression of the risk factors associated with HCV among 400 blood donors at FMC, Azare Northeast Nigeria

Characteristics	Total	HCV Pos. *N* (%)	COR (95% CI)	*p*-value	AOR (95% CI)	*p*-value
**History of blood donation**						
First time	107	3 (2.80)	2.08 (0.46–9.47)	0.342		
Repeated	293	4 (1.40)	1			
**Types of blood donor**						
Parental	128	2 (1.60)	1			
Volunteer	231	3 (1.30)	0.83 (0.14–5.03)	0.838		
Commercial donor	41	2 (4.90)	3.23 (0.44–23.69)	0.249		
**Surgical history**						
No	377	7 (1.90)	1			
Yes	23	0 (0.00)	0.00 (0.00)	0.999		
**History of blood transfusion**						
No	371	7 (1.90)	1			
Yes	29	0 (0.00)	0.00 (0.00)	0.999		
**Multiple sexual partners**						
No	337	6 (1.80)	1.12 (0.13–9.49)	0.915		
Yes	62	1 (1.60)	1			
**History of STD**						
No	392	7 (1.80)	1			
Yes	8	0 (0.00)	0.00 (0.00)	0.999		
**Piercing or tattoo**						
No	391	7 (1.80)	1			
Yes	9	0. (0.00)	0.00 (0.00)	0.999		
**Tribal mark**						
No	197	3 (1.50)	1			
Yes	203	4 (2.00)	1.30 (0.29–5.88)	0.734		
**Circumcision**						
No	5	0 (0.00)	1			
Yes	395	7 (1.80)	0.00 (0.00)	0.999		
**Sharp material**						
No	138	2 (1.40)	1			
Yes	262	5 (1.90)	1.32 (0.25–6.91)	0.740		

COR: Crude Odd Ratio; AOR: Adjusted Odds Ratio; 95% CI: 95% Confidence Interval

**Fig. 5:**
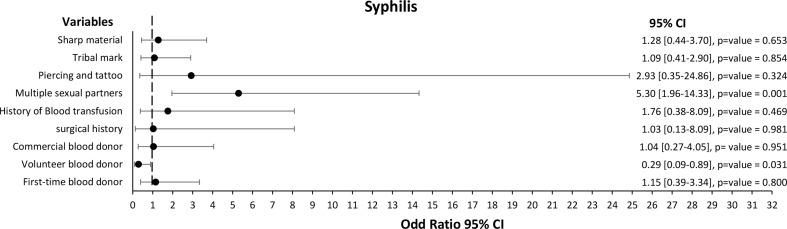
Forest plot of the risk factors associated with syphilis among 400 blood donors at FMC, Azare Northeast Nigeria

While for syphilis, the univariate analysis showed that syphilis was significantly linked with the voluntary type of blood donor [COR (95% CI): 0.29 (0.09–0.89)], multiple sexual partners [COR (95% CI): 5.30 (1.96–14.33)], and history of STDs [COR (95% CI): 267.00 (30.01-2382.92)] (Fig. 5).

The multivariate model was fitted, and only multiple sexual partners [AOR (95% CI): 4.35 (1.25–15.13)] and history of STDs [AOR (95% CI): 200.00 (20.46-1948.67)] were significantly associated with syphilis (Table 7).

**Table 7:** Univariate and multivariate logistic regression of the risk factors associated with syphilis among 400 blood donors at FMC, Azare Northeast Nigeria

Characteristics	Total	Syphilis Pos. *N* (%)	COR (95% CI)	*p*-value	AOR (95% CI)	*p*-value
**History of blood donation**						
First time	107	5 (4.70)	1.15 (0.39–3.34)	0.800		
Repeated	293	12 (4.10)	1			
**Types of blood donor**						
Parental	128	9 (7.00)	1			
Volunteer	231	5 (2.20)	0.29 (0.09–0.89)	0.031		
Commercial donor	41	3 (7.30)	1.04 (0.27–4.05)	0.951		
**Surgical history**						
No	377	16 (4.20)	1			
Yes	23	1 (4.30)	1.03 (0.13–8.09)	0.981		
**History of blood transfusion**						
No	371	15 (4.00)	1			
Yes	29	2 (6.90)	1.76 (0.38–8.09)	0.469		
**Multiple sexual partners**						
No	337	9 (2.70)	1		1	
Yes	63	8 (12.70)	5.30 (1.96–14.33)	0.001	4.35 (1.25–15.13)	0.021
**History of STD**						
No	392	10 (2.60)	1		1	
Yes	8	7 (87.50)	267.00 (30.01-2382.92)	0.001	230.00 (24.41-2184.73)	0.001
**Piercing or Tattoo**						
No	391	16 (4.10)	1			
Yes	9	1 (11.10)	2.93 (0.35–24.86)	0.324		
**Tribal mark**						
No	197	8 (4.10)	1			
Yes	203	9 (4.40)	1.09 (0.41–2.90)	0.854		
**Circumcision**						
No	5	0 (0.00)	1			
Yes	395	17 (4.30)	0.00 (0.00)	0.999		
**Sharp material**						
No	138	5 (3.60)	1			
Yes	262	12 (4.60)	1.28 (0.44–3.70)	0.653		

COR: Crude Odd Ratio; AOR: Adjusted Odds Ratio; 95% CI: 95% Confidence Interval

## Discussion

The WHO recommends blood collection from voluntary, non-remunerated donors as crucial for ensuring a safe and sufficient blood supply. These donors exhibit a reduced risk of TTIs compared to commercial donors and family replacements. This study documented the prevalence of various TTIs in prospective blood donors at the FMC Azare. In our study, the prevalence of TTIs was found to be 17.00%, surpassing the rates of 14.00% reported by Ndakotsu and Musa [16] in northwest Nigeria, 10.63% reported by Salawu [17] in South-west Nigeria, and 14.96% reported by Okoroiwu [9] in Calabar and 11.70% reported by Ndukwu and Chinedu-Madu [18] in Port Harcourt, which are both South-south regions of Nigeria. Similarly, lower seroprevalence was reported in other sub-Saharan areas, such as Somalia [19], Tanzania [20], Malawi [21], Ethiopia [3], and Uganda [22], which recorded 0.94%, 10.10%, 10.70%, 11.50%, 13.80% respectively. The high prevalence of TTIs identified in this study may lead to a significant decline in blood donors, raising public health concerns, particularly for individuals requiring long-term chronic care. Nevertheless, our discovery is below the prevalence rates reported by Nwankwo [23] in northwest Nigeria (19.30%) and 29.82% study reported by Nagalo [24] in other sub-Saharan Africa (Burkina Faso). The variation in prevalence could be attributed to disparities in the healthcare infrastructure throughout the study locations and the varied intensity of risk factors for acquiring TTIs in those settings.

The study found that the occurrence of HIV was 2.80%. The prevalence of this condition is comparable to the rates of 2.80% and 3.00% reported by Hassan [25] and Ndakotsu and Musa [16] in Kaduna and Sokoto, respectively, in the northwest region of Nigeria. Nevertheless, the percentage mentioned is lower than the 1.57% documented by Ejele and Ojule [26] in Port Harcourt, Southern Nigeria. Nagalo [24] found that Burkina Faso has a seroprevalence rate of 2.21%, which aligns with data obtained from other Sub-Saharan regions.

The gender distribution of HIV in this study reveals that males have a prevalence of 1.80%, markedly higher than the zero-prevalence reported in females. This finding agrees with the report of Okoroiwu [9] in Calabar, a southern region of Nigeria. The discrepancy may indicate possible gender-related variations in HIV exposure or risk behaviours among the study population. Risky behaviours demonstrated by males, such as participating in social activities outside of their residences and engaging in several sexual relationships, can also attributed to the findings [19]. So also, the elevated prevalence of HIV in male individuals who donate blood, in contrast to female blood donors, may be attributed to the predominance of males as blood donors in the study area [16].

The age categories of 28–37 exhibited a higher prevalence of HIV (3.80%) compared to the 18–27 (2.10%) age group, though the difference was not statistically significant. This tendency may indicate that persons in their late twenties to late thirties engage in behaviours that marginally elevate HIV risk. The outcome aligns with the findings of Mohammed [27] in Sudan, whose findings show that donors between the ages of 26–35 had a greater prevalence of HIV infection compared with other age ranges.

The elevated prevalence of HIV among adults aged 28– can be linked to their engagement in high-risk behaviors, such as having multiple sexual relationships and engaging in sexual activities that are not protected. The rate of HIV infection is highest among participants who have the primary school level of education (7.20%), and this difference is statistically significant compared to other education levels. There is no association between HIV infection and other sociodemographic factors, including the participant’s occupation, marital status, and residence.

HBV is a highly contagious disease that has affected around 2 billion individuals globally, with an estimated 400 million cases of chronic infection, and it is highly prevalent in sub-Saharan Africa and Asia [3]. The study found that the prevalence of HBV was 8.30%, and the study area was categorized as having a high endemic classification according to the WHO [28].

Our study’s seroprevalence of HBV is lower than the findings reported in other regions of Nigeria, such as Jos [29] and Taraba [30], which showed seroprevalence rates of 14.30% and 26.37%, respectively. In addition, similar findings were documented in other regions of Africa, including Cameroun [31] and Ghana [32], where the reported rates were 11.20% and 11.59%, respectively.

However, the seroprevalence of HBV observed in the present study exceeded the rates observed in different regions of Nigeria, such as Calabar [9] and Portharcourt [26], which were 4.1% and 1.67%, respectively. Similar results have been documented in other Sub-Saharan African nations, including Ethiopia [3] and Cameroun [33], where the rates were documented as 4.70% and 6.40% respectively. The current study has highlighted that the incidence of HBV among individuals who donate blood is higher (8.30%) compared to HIV (2.80%). The outcome aligns with the discovery made by Durowade [34] in Nigeria and Mremi [20] in Tanzania. HBV and HIV-infected individuals are permanently ineligible to donate blood, leading to a reduction in the pool of potential donors in the general population. These discrepancies may indicate variations in vaccination coverage and public health efforts among regions. The variation observed can also be attributed to differences in geographical location and diagnostic assays used, and certain sociocultural behaviours, such as piercings and tattoos, can also affect the prevalence rates.

The seroprevalence of HBV is greater among male participants compared to females, even though the disparity fails to attain statistical significance. The disparity may be attributed to more males serving as prominent blood donors in the research location than females. The variation in the prevalence of HBV concerning gender is consistent with findings of a study carried out in Ethiopia [35], which indicated a higher infection rate among males (3.70%) compared to females (1.00%). The age groups 18–27 (10.20%) and 28–37 (7.60%) have a higher seroprevalence of HBV than other age groups, although the variation failed to attain statistical significance. The highest seroprevalence of HBV in these age categories may be attributed to the varying risk behaviours observed.

The global prevalence of HCV infection is estimated to exceed 170 million [1]. Out of the 400 donors included in this study, 7 (1.80%) were infected with HCV. This percentage is comparable to the rates reported by Odenigbo [36] in Nnewi, South Eastern Nigeria. Nevertheless, this result is lower than the 3.40% documented by Bala [37] in Kano, the 6.10% reported by Dammulak [38] in Jos, and the 4.10% reported by Nwannadi [39] in Benue. Compared to some areas in Sub-Saharan Africa, Kamande [40] found a higher seroprevalence rate of 3.20% in Kenya, while Walana [41] reported a rate of 4.40% in Ghana.

Nevertheless, a lower prevalence of 1.10% [42] and 0.40% [3] was recorded in Nigeria and Ethiopia, respectively. The lower incidence of HCV compared to HBV in the present study may be attributed to HCV’s lower infectivity and its primary transmission modes, which include blood transfusion and needle sharing, which may not be shared in the study area. The study’s variation in HCV prevalence may be due to study design, geographical distribution, behavioural traits of participants in preventing viral hepatitis, and the duration of the studies [7, 43].

The seroprevalence of HCV was higher in the age categories of 28–37 and 18–27 years, with rates of 2.5 and 1.6, respectively, relative to other age categories. However, the observed disparity was insignificant. The increased prevalence of contracting HCV in young individuals may be attributed to a greater frequency of engaging in sexual activity with several partners within this age range. The distribution of HCV infection by gender revealed a prevalence of 1.80% among males and higher than 0.00% among females. This discovery is comparable to HCV seroprevalence among individuals who donate blood in Madagascar [44] and Ethiopia [43].

The research discovered that the overall occurrence of syphilis was 4.30%. The results are comparable to those of Angola [45] and Burkina Faso [24], which found prevalence rates of 4.40 and 3.90%, respectively. Nevertheless, the result derived from this study exceeds the 2.61% recorded by Salawu [17] in Ile Ife and the 3.6% reported by Chikwem [46] in Maiduguri, both within Nigeria. Similarly, there has been a lower prevalence in several African areas, including Kenya [40] and Ethiopia [3], where 1.20% and 0.10% were observed, respectively. However, Ghana has recorded a higher rate of 7.50%, according to Adjei [47]. The disparity in the occurrence of syphilis seen in this study, in comparison to prior studies, can be related to variances in the geographic distribution of the infection, as well as discrepancies in the accuracy and precision of screening tests. Differences in prevalence can also be associated with several factors, such as preventive measures, the efficacy of the screening program, donor selection, and healthcare accessibility [45]. The present study found that all the blood donors who had syphilis (2.10%) were entirely male, even though the difference was statistically insignificant. The higher percentage observed in the present study may be attributed to the fact that males are more susceptible to indulging in specific risky behaviours, including engaging in more than one sexual partner, which may increase their susceptibility to infection. The seroprevalence of syphilis was seen to be higher in the age category of 28–37 (8.30%) followed by those of age group 38–47 (1.90%), and the observed difference was statistically significance (*p* = 0.02). This result is consistent with prior research conducted by Alharazi [48] in Yemen. Young adults may partake in more dangerous sexual activities, such as having multiple sexual partners or using substances like alcohol or recreational drugs that can impair decision-making and result in unsafe sexual behaviour [49].

In general, the most often occurring TTIs was HBV, with a positive prevalence of 8.30%. This data exhibits similarities to previous reports conducted in Nigeria [34, 50] as well as in other countries, including Kenya [51, 52] and Ethiopia [5, 43]. The high prevalence of HBV can be linked to the virus’s strong infectivity potential and the low immunization level of the general population, leading to a high prevalence [53]. HBV is extremely infectious and can be easily spread from one person with the virus to another. HBV is found in all bodily fluids and secretions, such as blood, saliva, breast milk, urine, sweat, and semen. As a result, the virus can be transmitted through many channels, which seem to rely on the prevalence of the disease in a particular region [51].

The study found that the donor pool was predominantly male, accounting for 99.00% of the participants. This demographic pattern has been duplicated in previous studies conducted in some areas of Nigeria, such as Calabar [9] and Kano [23], which reported 98.7% and 98.0%, respectively. The same pattern has been documented in many regions around the globe, such as India [54], and Ethiopia [55], with respective recorded rates of 95.2, and 86.8. The higher representation of males in blood donation in this study can be attributed to the prevailing perception that males generally possess better health conditions than females, making them more eligible candidates for blood donation [56]. The temporary prohibition of female blood donation can also be attributed to physiological factors such as monthly blood loss through menstruation and pregnancy [57].

Notably, we noted a greater prevalence of co-infection among those aged 28–37. Similarly, the prevalence was higher in urban regions than rural ones and among individuals in business-related occupations than in other occupations. In contrast to our findings, a study conducted by Arshad [58] in Pakistan and Almugadam [10] in Sudan found that co-infection prevalence is higher in rural areas than in urban areas. In addition, our study revealed that the co-infection of HIV + Syphilis was the most common (1.0%), while the co-infections of HBV + Syphilis as well as HCV + Syphilis were the least common (0.5% each). The findings contradict the earlier studies conducted in Sudan [10] and China [59], indicating that the most common co-infection was HBV + Syphilis. These results collectively showed a presence of co-infection among the study participants in the study area.

There is a complete absence or scarcity of evidence, particularly in research, addressing the risk factors of TTIs among individuals who donated blood donors in the study area. These findings elucidate the presence of potential risk factors documented by blood donors who were found to be infected with HIV, HBV, HCV, and syphilis. The multivariate analysis indicated that the characteristics that had a statistically significant impact on HIV infection were engaging in multiple sexual partners and participating in commercial blood donation. The findings suggest that those who engage in multiple sexual activities have a 5.39-fold higher risk of contracting HIV (p-value = 0.02), whereas commercial blood donors have a 14.63-fold higher risk of infection (p-value = 0.01). The study highlights the high risk of HIV transmission among blood donors with multiple sexual partners, and this might be associated with behavioral risk factors in TTIs. Participating in multiple sexual relationships increases the likelihood of encountering an infected partner, especially without regular use of preventative measures. The biological efficacy of HIV transmission during unprotected sexual intercourse is exacerbated by restricted access to sexual health education and preventative treatments, as well as social stigma and condom utilization, increasing susceptibility to infection.

Regarding HBV infection, participants with a prior history of blood transfusion have a 4.33 times greater risk of acquiring HBV (p-value = 0.03). Additionally, individuals who have undergone tattooing or piercing have a 5.023 times greater chance of infection (p-value = 0.03). In contrast, no association was found between HCV and related risk factors. Concerning syphilis infection, engaging in several sexual partners is linked to a 4.348-fold increase in risk (p-value = 0.02), while having a previous history of sexually transmitted diseases is connected with a 230-fold increase in risk (p-value = 0.01). The observed high prevalence of HBV and syphilis among blood donors with multiple sexual partners may also be largely linked to behavioral risk factors. High-risk sexual behaviors, such as multiple sexual partnerships, increase the likelihood of exposure to infected individuals, especially without consistent use of barrier protection methods like condoms. The result agrees with the finding of Liu [49] in Chengdu, China, which reported that blood donors who have multiple sexual partners have a seven-fold higher risk of contracting syphilis. The current study indicates that having several sexual partners is a substantial risk factor for HIV and syphilis infection, as this variable is statistically significant in multivariate analysis. As sexual awareness becomes increasingly liberal, numerous individuals utilize mobile applications to seek sexual partners, resulting in a rise in the prevalence of multiple sexual partners. These findings suggest the need to strengthen the screening and employ state-of-the-art techniques and advanced kits for blood donors before donation.

The present study found that voluntary donors were the predominant type of donors, followed by parental or family donors, with commercial donors being the least prevalent. This finding offers additional substantiation for the WHO report that there has been a significant increase in voluntary blood donation from 2008 to 2018 in different continents, with an 81% increase in Africa [2]. The result also agrees with what Ntawuyamara [60] reported in Burundi, which shows that most blood donors were voluntary. However, this discovery contradicts the previous findings by Damulak [61] in Jos, North Central Nigeria, which said that commercial donors dominate the blood donation process since most of the blood is donated by commercial donors. Their findings suggested that the quantity of blood donated voluntarily in Nigeria has consistently decreased over time, and this decline can be attributed to logistical and organizational challenges faced by the Nigerian national blood transfusion service. The difference in results can be ascribed to the location of the studies. The research in Jos was conducted at the NBTS centre, while ours was conducted at a blood bank within a healthcare facility. In Nigeria, national blood transfusion centres regularly organize blood drives to encourage voluntary blood donation. This contrasts with healthcare facility-based blood banks, which primarily rely on voluntary donors who walk in.

Although voluntary donors are the majority of donors in the present investigation, commercial donors recorded the highest occurrence of TTIs compared to other blood donors. This discovery aligns with the findings of Okoroiwu [9] in Calabar, a city in the southern region of Nigeria. The study revealed a greater frequency of TTIs among commercial blood donors, while voluntary donors had the least prevalence. This discovery provides more evidence to support the previous assertion by the WHO that volunteer donors have a lower likelihood of transmitting TTIs compared to family replacement and commercial donors [2]. Individuals facing serious economic difficulties are less inclined to disclose their proper health condition, and the potential monetary compensation may attract donors willing to take greater risks. The relatively low seroprevalence of TTIs observed in the present study might be attributed to the type of kit utilized.

Pre-donation education is crucial for blood safety and educating potential donors about the donation process, eligibility criteria, and TTI risks. This reduces anxiety and encourages risk disclosure, as studies have shown that well-informed blood donors are more likely to comply with eligibility requirements [62]. Donor selection and strict screening criteria can significantly minimize the risk of TTIs. Studies conducted in some African countries such as Ethiopia [8], Somalia [19], and Eritrea [63] report a decline in the seroprevalence of TTIs, including HIV, HBV, HCV, and syphilis. The studies attributed these declines to strict donor selection and comprehensive screening protocol. In addition, excluding or deferring individuals with potential exposure to high-risk behaviour is also critical in minimizing the risk of TTIs. Several studies have indicated that blood donors who deferred their blood donation due to high-risk behaviour have a higher prevalence of TTIs compared to those who did not defer [64, 65]. In our study area, where TTIs remain a public health challenge, implementing these strategies will ensure the safety of blood recipients and foster trust and confidence in the blood donation system.

### Study limitations

The study’s single-centre data from the Blood Bank of FMC Azare may limit its generalizability to larger populations. Self-reported data may also introduce biases related to recall and social desirability. The limited representation of female participants among female donors constrains the study’s generalizability across genders. This study may be limited by the screening technique employed for TTIs, which may lack the sensitivity or specificity of more sophisticated methods such as enzyme immunoassays (EIA), chemiluminescent immunoassays (CLIA), or nucleic acid testing (NAT). These advanced techniques can address the underestimation issue, which might arise in the present study due to a window period, providing the actual burden of TTIs infection in the study area. Irrespective of the highlighted limitation, the primary strength of the study was the availability of abundant data to evaluate the sociodemographic and risk factors associated with TTIs, complemented by a substantial sample size (*n* = 400), which provided insight into the prevalence of TTIs in the study area.

## Conclusion

The investigation at Federal Medical Centre Azare found a 17.0% incidence of TTIs in blood donated, with HBV being the most common. Commercial donors, multiple sexual partners, prior transfusion, and piecing or tattoos were significantly associated with TTIs. The gender discrepancy in blood donation is significantly influenced by societal conventions, highlighting the challenges in achieving more equitable participation. The occurrence of TTIs in commercial blood donors underscores the need for stringent selection criteria and thorough screening. We recommend the nationwide adoption of advanced screening technologies like NAT in transfusion protocols and strengthened public health measures to reduce TTIs prevalence. Policymakers should also promote voluntary blood donation through public awareness programs to ensure a safe and sufficient blood supply.

## Electronic supplementary material

Below is the link to the electronic supplementary material.

Supplementary Material 1

## Data Availability

All materials are within the manuscript; any additional data will be available upon reasonable request from the corresponding author.

## Abbreviations

HBV: Hepatitis B virus
HCV: Hepatitis C virus
HIV: Human immunodeficiency virus
TTIs: Transfusion-transmissible infections
STD's: Sexual Transmitted Diseases
WHO: World Health Organization
EIA: Enzyme Immunoassays
CLIA: Chemiluminescent Immunoassays
NAT: Nucleic acid testing
NBTS: National Blood Transfusion Service
FMC: Federal Medical Centre
COR: Crude Odd Ratio
AOR: Adjusted Odds Ratio
CI95%: 95% Confidence Interval
