# Supplementary material for: Seroprevalence of HIV, HBV, HCV and Syphilis among blood donors in a Nigerian tertiary medical centre
Source: BMC Infect Dis. 2025 Apr 30;25:638. doi: 10.1186/s12879-025-11024-z (PMC12044881; doi:10.1186/s12879-025-11024-z)
Supplement: Supplementary file 1 — Supplementary Material 1 [file 12879_2025_11024_MOESM1_ESM.docx]

**Questionnaire**

**Questionnaire No:**

Seroprevalence of HIV., HCV, HBV And Syphilis and The Associated Risk Factors Among Blood Donors in a Tertiary Medical Centre in Nigeria

*(Tick the letter corresponding to your answer.)*

**Sociodemographic Characteristics**

| **Gender**  a. Male  b. Female | **Marital Status**  a. Single  b. Married  c. Divorced  d. Widow | **Occupation**  a. Civil servant  b. Student  c. Farmer  d. Business  e. Teaching  f. Driving  g. Housewife |
| --- | --- | --- |
|  |  |  |
| **Age:**  a. 18-27 years  b. 28-37 years  c. 38-47 years  d. > 48 years | **Level of Education**  a. Cannot read and write  b. Primary Education  c. Secondary Education  d. Tertiary Education | **Residence**  a. Urban  b. Rural |

**Factors Associated with HIV, HCV, HBV And Syphilis**

| **History of Blood Donation**  a. Fresh  b. Repeated | **Multiple Sexual Partners**  a. No  b. Yes | **Circumcision**  a. No  b. Yes |
| --- | --- | --- |
|  |  |  |
| **Types of Blood Donor**  a. Parental  b. Volunteer  c. Commercial | **History of STD**  a. No  b. Yes | **Use of Sharp Materials**  a. No  b. Yes |
|  |  |  |
| **Surgical History**  a. No  b. Yes | **Piecing/Tattoo**  a. No  b. Yes |  |
|  |  |  |
| **History of Blood Transfusion**  a. No  b. Yes | **Tribal Marks**  a. No  b. Yes |  |

**Clinical results:**

HIV__________, HCV__________, HBV____________ and Syphilis__________________
